# The complete chloroplast genome of *Theobroma grandiflorum*, an important tropical crop

**DOI:** 10.1080/23802359.2019.1693291

**Published:** 2019-11-20

**Authors:** Ying-Feng Niu, Shu-Bang Ni, Jin Liu

**Affiliations:** Yunnan Institute of Tropical Crops, Xishuangbanna, China

**Keywords:** Chloroplast genome, sequence, *Theobroma grandiflorum*

## Abstract

*Theobroma grandiflorum* (Willd. ex Spreng.) K. Schum., an economically important tree in the family Malvaceae, is native to the Amazonian region of South America and it is widely cultivated as a tropical crop. Herein, we report the complete chloroplast genome of *T. grandiflorum*. The size of the complete chloroplast genome of *T. grandiflorum* is 160,606 bp with 36.88% GC content, which includes a pair of inverted repeat regions (IRs) of 25,496 bp separated by a large single-copy region (LSC) of 89,429 bp and a small single copy (SSC) region of 20,185 bp. A total of 126 genes were annotated, of which 82 were protein-coding genes, 36 were transfer RNA (tRNA) genes, and 8 were ribosomal RNA (rRNA) genes. A maximum-likelihood (ML) analysis supported a close relationship between *T. grandiflorum* and *T. cacao.* This study will provide useful information for further phylogenetic and evolutionary analysis of Malvaceae.

*Theobroma grandiflorum* (Willd. ex Spreng.) K. Schum., an economically important tree in Malvaceae, is native to the Amazonian region of South America (Fischer et al. 1995). The genus *Theobroma* is divided into six sections, *Andropetalum*, *Glossopetalum*, *Oreanthes*, *Rhytidocarpus*, *Telmatocarpus*, and *Theobroma*, and includes 22 species that are distributed in the rain forests of South America and Mexico (Silva et al. 2004), but only two species (*T. cacao* L. and *T. grandiflorum*) were widely cultivated as tropical crops (Santos et al. 2012).

The fruit of *T. grandiflorum* is used as feedstock for multiproduct biorefinery, resulting in pasteurized pulp, antioxidant extract, biofertilizer, biogas, seed oil, essential oil, ethanol, and polyhydroxybutyrate (Cerón et al. 2015). The pulp of *T. grandiflorum* fruit is consumed in juices, ice creams, or bakery fillings (Fischer et al. 1995). The seeds contain high amounts of fat and may be used in food products and in a variety of cosmetics (Pugliese et al. 2013).

Although *T. grandiflorum* is an important tropical crop, reports on its genetics and genomics are limited (Kuhn et al. 2010), and its complete chloroplast genome has not yet been reported. Chloroplast DNA contains a wealth of genetic information, and its sequences can provide useful molecular markers for genetic studies (Argout et al. 2011; Liu et al. 2018). In this study, the complete chloroplast genome of *T. grandiflorum*, with material obtained from Xishuangbanna Tropical Flowers and Plants Garden as rooted plants (the geospatial coordinates are N 22.00958325 and E 100.78632821), was first reported and characterized. *T. grandiflorum*. Genomic DNA was isolated from healthy young leaves using a Dneasy Plant Mini Kit (Qiagen) and stored in the ultra-low temperature specimen library of YITC (specimen accession number: YITC-2019-FZ-T-001). The GS Titanium Library Preparation Kit and the GS Junior Titanium Sequencing Kit (Roche 454 Life Sciences, Branford, Connecticut, USA) were used to generate a shotgun library of genomic DNA and sequencing on the Roche/454 system (Roche 454 Life Sciences). Sequencing data were assembled using the CLC Genomic Workbench v3.6 (http://www.clcbio.com) and the chloroplast genome was annotated using DOGMA (Wyman et al. 2004) with manual correction. The complete chloroplast genome sequence and gene annotations of *T. grandiflorum* were submitted to GenBank under accession number MN562270.

The size of complete chloroplast genome of *T. grandiflorum* is 160,606 bp, which includes a pair of inverted repeat regions (IRs) of 25,496 bp separated by a large single-copy region (LSC) of 89,429 bp and a small single copy (SSC) region of 20,185 bp. The composition of the four bases in the circular chloroplast genome is 31.04% A, 32.08% T, 18.07% G, and 18.81% C, and the GC content of the entire *T. grandiflorum* chloroplast genome is 36.88%. A total of 126 genes were annotated in the *T. grandiflorum* chloroplast genome, of which 82 were protein-coding genes, 36 were transfer RNA (tRNA) genes, and 8 were ribosomal RNA (rRNA) genes. The functions of the protein-coding genes in the *T. grandiflorum* chloroplast genome include photosystem I, photosystem II, cytochrome b/f complex, ATP synthase, NADH dehydrogenase, RubisCO large subunit, RNA polymerase, ribosomal proteins, and other genes.

Phylogenetic analysis based on complete chloroplast sequences was performed using 21 Malvaceae species and *Vatica mangachapoi* (Dipterocarpaceae) as the outgroup (Figure 1). The sequences were aligned using MAFFT (Katoh and Standley 2013), and the maximum-likelihood (ML) analysis was performed using MEGA7 (Kumar et al. 2016) with 1000 bootstrap replicates. The results supported a close relationship between *T. grandiflorum* and *T. cacao*, which has a larger chloroplast genome than *T. grandiflorum* but contains fewer genes. This study provides useful information for further phylogenetic and evolutionary analyses of Malvaceae.

**Figure 1.**
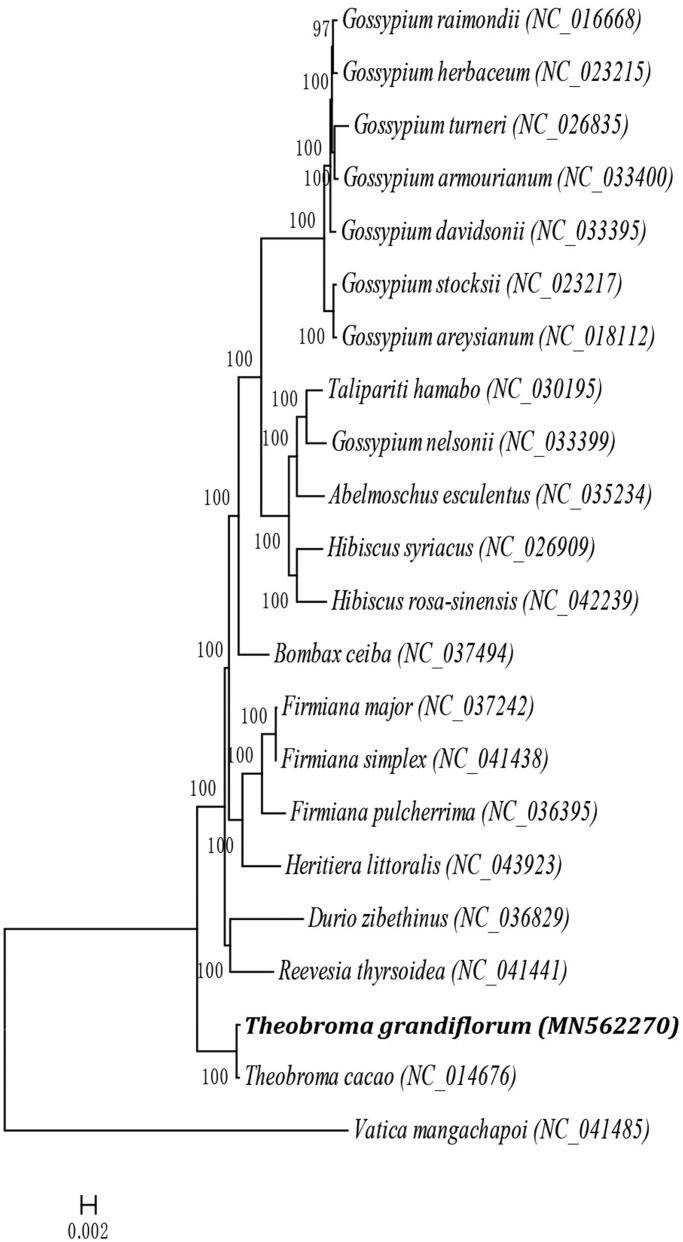
Maximum-likelihood phylogenetic tree of *T. grandiflorum* with 21 species in the family of Malvaceae and Dipterocarpaceae, order Malvales based on complete chloroplast genome sequences. Numbers in the nodes are bootstrap values from 1000 replicates. Bootstrap values are shown above the nodes. The chloroplast genome accession number for tree construction is listed as follows: *Gossypium areysianum* (NC_018112), *Gossypium armourianum* (NC_033400), *Gossypium davidsonii* (NC_033395), *Gossypium herbaceum* (NC_023215), *Gossypium raimondii* (NC_016668), *Gossypium stocksii* (NC_023217), *Gossypium turneri* (NC_026835), *Talipariti hamabo* (NC_030195), *Gossypium nelsonii* (NC_033399), *Abelmoschus esculentus* (NC_035234), *Hibiscus rosa-sinensis* (NC_042239), *Hibiscus syriacus* (NC_026909), *Bombax ceiba* (NC_037494), *Firmiana major* (NC_037242), *Firmiana simplex* (NC_041438), *Firmiana pulcherrima* (NC_036395), *Heritiera littoralis* (NC_043923), *Durio zibethinus* (NC_036829), *Reevesia thyrsoidea* (NC_041441), *Theobroma cacao* (NC_014676), *Theobroma grandiflorum* (MN562270), *Vatica mangachapoi* (NC_041485).
